# Prognostic significance of the number of lymph nodes in elective neck dissection for tongue and mouth floor cancers

**DOI:** 10.1590/S1808-86942012000200005

**Published:** 2015-10-20

**Authors:** Ali Amar, Helma Maria Chedid, Abrão Rapoport, Claudio Roberto Cernea, Rogério Aparecido Dedivitis, Otávio Alberto Curioni, Lenine Garcia Brandão

**Affiliations:** a PhD in Medicine – Graduate Program in Otorhinolaryngology and Head and Neck Surgery – Federal University of São Paulo (UNIFESP). Surgeon at the Otorhinolaryngology and Head and Neck Surgery Department – Heliópolis Hospital, São Paulo – SP; b MSc in Sciences – Heliópolis Hospital, São Paulo, SP. Surgeon at the Otorhinolaryngology and Head and Neck Surgery Department – Heliópolis Hospital, São Paulo – S P; c Senior Associate Professor – Department of Surgery – Medical School of the University of São Paulo – USP, São Paulo, SP. Surgeon at the Otorhinolaryngology and Head and Neck Surgery Department – Heliópolis Hospital, São Paulo – S P; d Associate Professor – Department of Head and Neck Surgery – Medical School of the University of São Paulo USP, São Paulo, SP; e MD. Senior Associate Professor – FundaçãoLusíada – UNILUS; f PhD in Medicine – Graduate Program in Pathology – Medical School of the University of São Paulo. Head of the Department of Head and Neck Surgery – Heliópolis Hospital, São Paulo –SP); g Full Professor – Department of Head and Neck Surgery – Medical School of the University of São Paulo – USP, São Paulo, SP. Departamentos de Cirurgia de Cabeça e Pescoço e Otorrinolaringologia do Hospital Heliópolis e de Cirurgia de Cabeça e Pescoço da Faculdade de Medicina da USP, São Paulo/SP

**Keywords:** carcinoma, squamous cells, lymph nodes, mouth neoplasms, neck dissection

## Abstract

The presence of metastatic lymph nodes is a relevant aspect in the treatment of head and neck cancer, bringing about a 50% reduction in survival.

**Objective:**

To assess the number of lymph nodes removed in the neck dissection and their relationship with the prognosis.

**Methods:**

A retrospective study involving 143 patients with tongue and mouth floor epidermoid carcinoma, which histological exam showed no lymph node metastases. Among those, 119 were males and 24 females, with mean age of 54 years. As to the primary tumor site, 65 were in the tongue and 78 in the mouth floor. T stage distribution was of four T1, 84 T2, 36 T3 and 19 T4. We carried out 176 neck dissections, unilateral in 110 cases and bilateral in 33. Of these, 78 were radical and 98 selective. The patients were broken down into three groups, according to the 33 and 66 percentiles of the number of lymph nodes resected.

**Results:**

The mean number of resected lymph nodes was 27; 24 in selective dissections and 31 in the complete ones. We did not have statistically significant differences when associated to the T and N stages.

**Conclusions:**

The larger number of lymph nodes dissected in the neck dissection identifies the group of better prognoses among pN0 cases.

## INTRODUCTION

Neck dissection is a standardized procedure, being indicated for staging and treating regional metastases of malignant tumors of the upper air and digestive tracts. Metastatic lymph nodes represent one of the most relevant aspects associated with treatment, characterizing an advanced clinical stage (stages III or IV), and it is associated with a 50% reduction in survival^1^. Although the number of lymph nodes removed is prognostically relevant in other areas of the body (colon and breast), very few studies have assessed their meaning in head and neck tumors^2^, ^3^, ^4^, ^5^, ^6^. Moreover, the number of lymph nodes removed may also be an indicator of how radical the dissection is as a staging method. It is estimated that approximately 7% of pN0 cases have micrometastasis unidentified in routine sections^7^. We did not find any disease-free survival differences in association with the number of lymph nodes dissected^8^.

The goal of the present study is to assess the number of lymph nodes removed in neck dissections and investigate their relationship with the locoregional control and survival prognosis.

## METHODS

This project was approved by the Ethics in Research Committee of the institution where the patients were treated.

We reviewed the charts from the patients submitted to neck dissection because of tongue or mouth floor epidermoid carcinoma between January of 1985 and December of 2007. As inclusion criteria, we enrolled all the patients who were consecutively submitted to surgical treatment and neck dissection, without lymphatic metastases upon pathology exam. The exclusion criterion was: having been submitted to some kind of prior treatment for the tumor. We selected 143 patients, of whom the histopathological exam confirmed that there were not lymph node metastases (pN0). They were all clinically staged. Of these patients, 119 were men and 24 were women, with a mean age of 54 years (22 to 78). All the patients had epidermoid carcinoma, 65 with a primary tumor in the tongue and 78 cases of mouth floor tumors. As to the T staging, four were T1; 84 T2; 36 T3 and 19 were T4. Staging was elective in 110 cases and therapeutic in 33 (false-positives). We carried out 110 unilateral dissections and 33 bilateral, making up a total of 176 dissections, 78 radical-modified and 98 were selective.

The lymph nodes were divided in levels immediately after the surgery, by one of the team's surgeon in the operating theater, and sent for histological test, separated and identified in the proper levels. Histopathology was carried out with hematoxylin and eosin.

We assessed the number of lymph nodes, according to the type of dissection, gender, age and staging. The difference between the groups was evaluated using the Mann-Whitney and Kruskal-Wallis tests. The patients were broken down into three groups, according to percentiles 33 and 66 of the number of lymph nodes resected; and we assessed locoregional control and disease-free survival using the Kaplan-Meier method, and the difference between the groups using the *log-rank test*. We deemed significant those differences with alpha error lower than 5%.

## RESULTS

The mean number of lymph nodes was 27; 24 were selective dissections and 31 were radical. The median was 25 lymph nodes per dissection, with a minimum of seven and a maximum of 72, being the same value both for males and females.

Local, isolated or associated recurrences, happened in 34 patients. Regional recurrences were seen in 24 cases, being isolated in nine of these. In 22 patients we also diagnosed a second primary tumor. Distant recurrences happened in seven patients; notwithstanding, it was isolated in only two.

**Figure 1:**
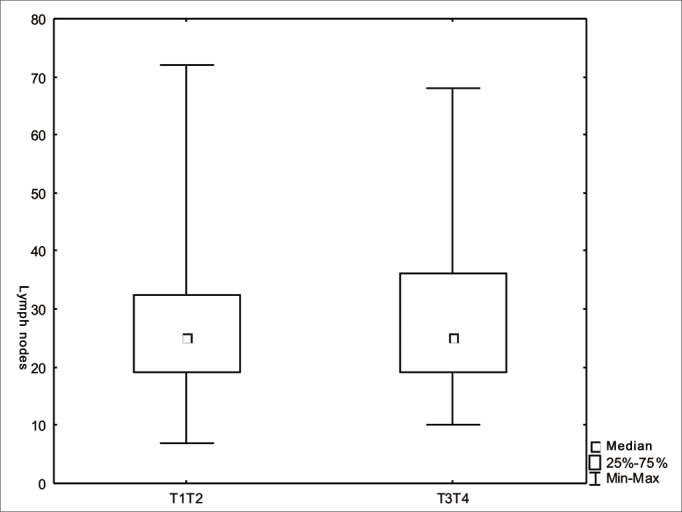
Number of lymph nodes according to stage T (*p*=0.45).

**Figure 2:**
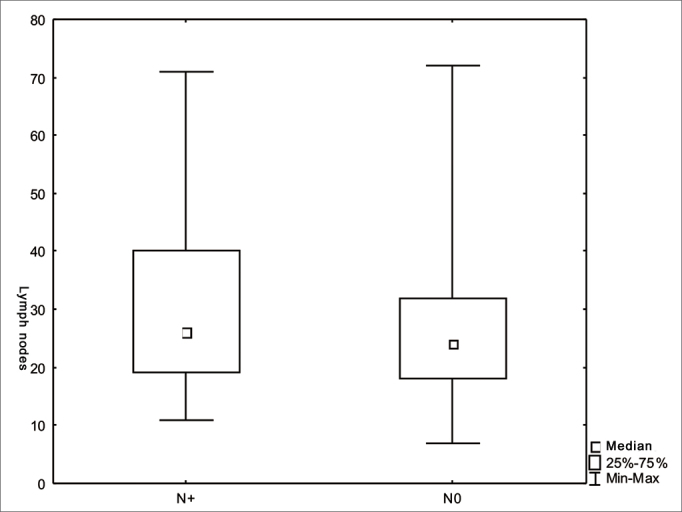
Number of lymph nodes according to stage N (*p*=0.08).

**Figure 3:**
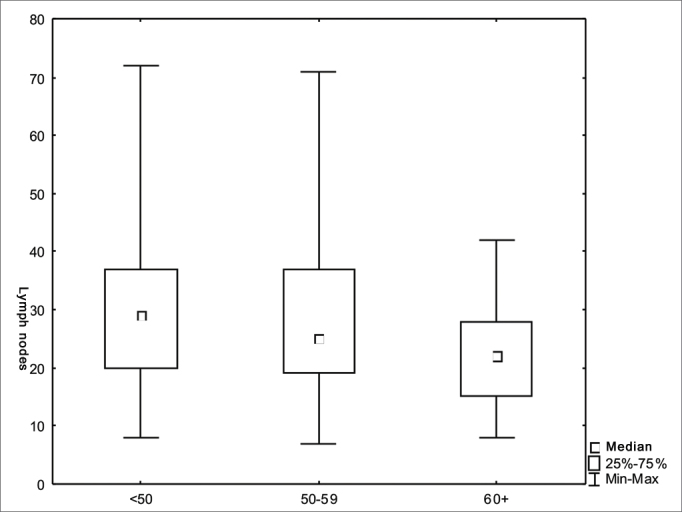
Number of lymph nodes according to the age range (*p*=0.01).

We did not find significant differences regarding the number of lymph nodes vis-à-vis the T and N staging (Figures 1 and 2). There was an inverse relation between the number of lymph nodes and patient age range (Figure 3).

Disease-free survival in two and five years were 86% and 59%, respectively, among the patients with 30 or more lymph nodes upon dissection; while among the patients with more than 30 lymph nodes, survival was 70% and 59%, respectively (*p*=0.07).

**Figure 4:**
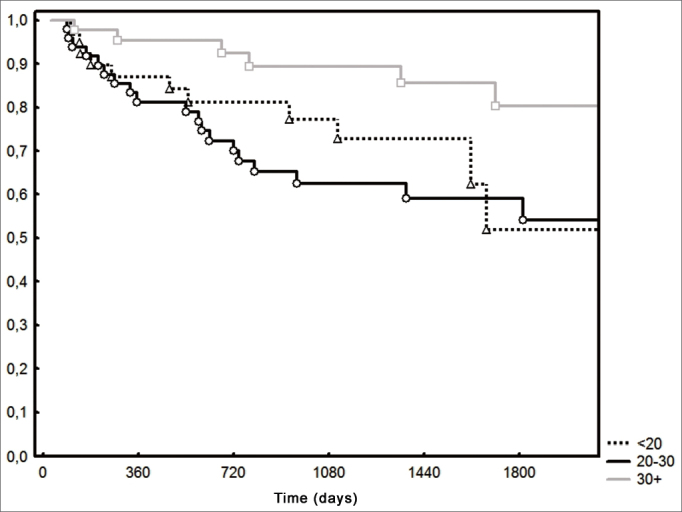
Loco-regional control according to the number of lymph nodes (*p*=0.02).

Locoregional control was higher (*p*=0.02) in patients with 30 or more lymph nodes (Figure 4). As far as the T stage is concerned, the 2-year locoregional control was 80% both in T1 and T2 patients as well as in the T3 and T4 (*p*=0.77) patients. As far as resection margins are concerned, they were compromised or exiguous in 20 cases; the two-year locoregional control was 84% when free and 66% when compromised (*p*=0.77).

## DISCUSSION

There is a recent report that the number of lymph nodes found in the neck dissection is a prognostic factor of mouth cancer^2^. Patients with less than 18 lymph nodes had lower disease-specific and global survivals, confirmed in the multivariate analysis^2^. In the present study, the patients with 30 or more lymph nodes had a better locoregional control and higher two-year survival; however, there was no significant difference associated to disease-free survival. Nonetheless, this finding seems to have little relevance vis-à-vis clinical application, in which the cutting point has a large likelihood of corresponding to a statistical artifact. Similarly to what was reported by Ebrahimi et al.^2^, there was an overlapping of results among the patients grouped in the other age ranges. Notwithstanding, this brings back an important issue concerning the role of regional lymph nodes in cancer control. The number of lymph nodes removed in the neck dissection seems to be more dependent on the identification method utilized with the surgical specimen than the neck dissection technique, since it is strenuously standardized and already established among specialists. Thus, we believe that the number of lymph nodes is not associated with the quality of the neck dissection approach, which would be a very simplistic explanation for the number of recurrences among patients with few lymph nodes dissected. One must also consider that the neck is an infrequent site of isolated recurrence and lymph node metastases reveals an aggressiveness phenotype which is not only regional. Agrama et al. ^3^ reported a higher probability of finding metastases when more than 20 lymph nodes are dissected. Moreover, there is an expectation that approximately 7% of the pN0 cases have unidentified micrometastases in routine sections^7^. Thus, one possible explanation for the worse disease control among patients with few lymph nodes found would be the high rate of false-negatives. This possibility could explain the regional recurrences in this group; nonetheless, among the nine cases of patients who developed isolated regional recurrences, all had 20 or more lymphnodes upon neck dissection.

Notwithstanding the comparison of the number of lymph nodes between selective and radical dissections were not significant, there was a trend towards a difference. It is supposed that radical dissections will yield more lymph nodes than the selective ones, with a trend towards significant differences, which could impact the results. However, despite the bias in including dissections with a different extension – which impacts the number of lymph nodes dissected, it is expected to have similar regional control among patients submitted to selective or completeneck dissections, especially when all the cases are pN0^9^. We did not find differences in the number of lymph nodes in relation to the T stage. Surprisingly, the T stage was not significant as to the prognoses of these patients.

One aspect which must help identify a larger number of lymph nodes may be their reactivity, leading to the increase in size. There was a marginally significant relationship between the presence of palpable lymph nodes (N+pN0) and the number of lymph nodes removed. Favoring such hypothesis, we observed an inverse relationship between the number of lymph nodes and the age of the patients, explained by the lower immune response among the older patients^3^. The lymph node reactivity relationship with the prognosis was shown in different studies starting in the 50's, although not important enough to justify its routine use in the individual's prognosis^10^. The assessment of 26 pN0 patients treated for oral cavity cancer showed germ center patterns or a lymphocytic predominance in 27% in the group which failed control and 73% in the disease-free group of patients. On the other hand, collectively, the normal, sinus histiocytosis and lymphocytic depletion patterns were seen in 73% in the group which failed control, which seems to show a prognostic importance^11^. 3,648 lymph nodes from 105 patients were assessed in one study with laryngeal cancer patients. Sinus histiocytosis, paracortical hyperplasia and follicular hyperplasia reactivity patterns were not associated with recurrence or mortality; nonetheless, the cell immunity stimulus associated with the neck lymph node hyperplasia was significantly related to a better global five-year survival in pN0 patients^12^.

Although the locoregional control was better in patients with 30 or more lymph nodes, the five-year disease-free survival was similar, stressing the frequency of a second primary tumor, which happened in 22 cases (15%). Other authors also failed to find disease-free survival differences in relation to the number of lymph nodes dissected^8^. In the univariate analysis, only the number of lymph nodes was statistically significant in relation to the locoregional control.

There are many factors with prognostic impact on this group of patients, such as primary tumor staging, number of pathologically positive lymph nodes, adjuvant treatment (post-operative radio or chemotherapy), perineural invasion, angiolymphatic invasion, surgical margins situation, and others; we recommend future studies using the multivariate analysis in order to better appreciate the results obtained.

It is obvious that neck dissections must remove the most lymph nodes from the selected chains, as well as identify them, thus enabling proper staging and adjuvant treatment. The greater number of lymph nodes collected in the dissection may identify a group with better prognosis in pN0 cases, with the need for multivariate analysis studies. Such importance is even clearer when we find a trend towards a better disease-free survival in the multivariate analysis of the group with 30 or more lymph nodes dissected (*p*=0.07).

## CONCLUSIONS

The larger number of lymph nodes seen in the surgical specimen is associated with a better locoregional control; nonetheless, without statistical significance concerning a better survival.
